# Phylogenetic Analysis of the Neks Reveals Early Diversification of Ciliary-Cell Cycle Kinases

**DOI:** 10.1371/journal.pone.0001076

**Published:** 2007-10-24

**Authors:** Jeremy D. K. Parker, Brian A. Bradley, Arne O. Mooers, Lynne M. Quarmby

**Affiliations:** 1 Department of Molecular Biology and Biochemistry, Simon Fraser University, Burnaby, British Columbia, Canada; 2 Department of Biological Sciences, Simon Fraser University, Burnaby, British Columbia, Canada; University of British Columbia, Canada

## Abstract

**Background:**

NIMA-related kinases (Neks) have been studied in diverse eukaryotes, including the fungus *Aspergillus* and the ciliate *Tetrahymena*. In the former, a single Nek plays an essential role in cell cycle regulation; in the latter, which has more than 30 Neks in its genome, multiple Neks regulate ciliary length. Mammalian genomes encode an intermediate number of Neks, several of which are reported to play roles in cell cycle regulation and/or localize to centrosomes. Previously, we reported that organisms with cilia typically have more Neks than organisms without cilia, but were unable to establish the evolutionary history of the gene family.

**Methodology/Principle Findings:**

We have performed a large-scale analysis of the Nek family using Bayesian techniques, including tests of alternate topologies. We find that the Nek family had already expanded in the last common ancestor of eukaryotes, a ciliated cell which likely expressed at least five Neks. We suggest that Neks played an important role in the common ancestor in regulating cilia, centrioles, and centrosomes with respect to mitotic entry, and that this role continues today in organisms with cilia. Organisms that lack cilia generally show a reduction in the number of Nek clades represented, sometimes associated with lineage specific expansion of a single clade, as has occurred in the plants.

**Conclusion/Significance:**

This is the first rigorous phylogenetic analysis of a kinase family across a broad array of phyla. Our findings provide a coherent framework for the study of Neks and their roles in coordinating cilia and cell cycle progression.

## Introduction

The NIMA-related family of serine/threonine kinases (Neks) are widespread among eukaryotes. They are defined by similarity in their N-terminal catalytic domains to the founding member, Never In Mitosis A from the fungus *Aspergillus* [1], [2]. NIMA, the sole Nek in the *Aspergillus* genome, plays multiple roles in cell cycle progression and localizes to the fungal equivalent of the centrosome, the spindle pole body [1], [3]. There are 11 NIMA-related kinase (Nek) genes in the human genome. Those that have been studied appear to have cell cycle-related functions and some localize to centrosomes [4]–[11].

Centrosomes are typically located near the nucleus in the centre of the cell, where they serve as organizers of the microtubular cytoskeleton during both interphase and mitosis [12]. In addition to their well-established role as microtubule organizers, centrosomes serve as important scaffolds for the integration of multiple signaling pathways that coordinate cell cycle progression [13]. Of the centrosomal Neks, only for Nek2 has a function been clearly defined. Nek2 phosphorylates proteins which physically link centrioles, the microtubule-based structures at the core of centrosomes [14]–[16].

In mammalian cells, the elder centriole of the centrosomal pair often directly nucleates a cilium. Cilia have functions in both motility and sensory signaling [17]. The recent demonstration that flies without centrioles develop normally but then die due to multiple sensory defects [18] suggests that the most important function of centrioles may be to nucleate sensory cilia, at least in some lineages. An indication of the ciliary function of some Neks derives from their association with polycystic kidney diseases, which are caused by defective ciliary signaling [19]: The causative mutations in two mouse models of polycystic kidney diseases are in the mNek1 and mNek8 genes [20], [21]. We have shown that Nek8 is ciliary [11] and mutations that affect ciliary localization are associated with a rare form of a cystic kidney disease that affects children, Nephronophthisis type 9 [22]. Although the functions of Neks in mammalian cilia are unknown, it has been established that Neks in *Chlamydomonas* and *Tetrahymena* regulate ciliary length and/or disassembly [23]–[26]. We have previously noted a correlation between the number of Neks a eukaryotic organism expresses, and the presence of ciliated cells which re-enter the cell cycle in that organism [2]. Together, these observations have led us to propose that Neks evolved with centrosomes and serve to coordinate ciliary and cell cycle functions [2], [27].

The breadth of organisms in which Neks are found and the lack of sequence conservation outside the kinase domains has meant no large-scale phylogenetic analysis has been possible via traditional methods. Many authors refer to NIMA as “ancestral” to the mammalian Neks, yet no eukaryotic phylogeny would ever place *Aspergillus* as an ancestor to mammals. The assumption by these authors is that the last common ancestor of Fungi and Metazoa had one Nek, similar to NIMA, and this gene underwent an expansion in Metazoa and especially in mammals. This conflicts with previously published phylogenies [26], [28] which suggest that there are orthologies between mammalian Neks and Neks in both *Chlamydomonas* and *Tetrahymena*. However, these earlier Nek phylogenies had few taxa and poor support at many nodes. We set out to establish a more complete Nek family tree, by sampling distantly-related phyla, by using Bayesian inference methods, and by testing specific hypotheses in a Bayesian framework. To our knowledge, no similar analyses have been published for a eukaryotic kinase family. Our results suggest that the last common ancestor of the eukaryotes, which must have been a ciliated cell able to divide [29], had at least five Neks. This finding and new cell biological data further strengthen the three-way bond between Neks, cilia/centrosomes, and the cell cycle.

## Materials and Methods

**Figure 1:**
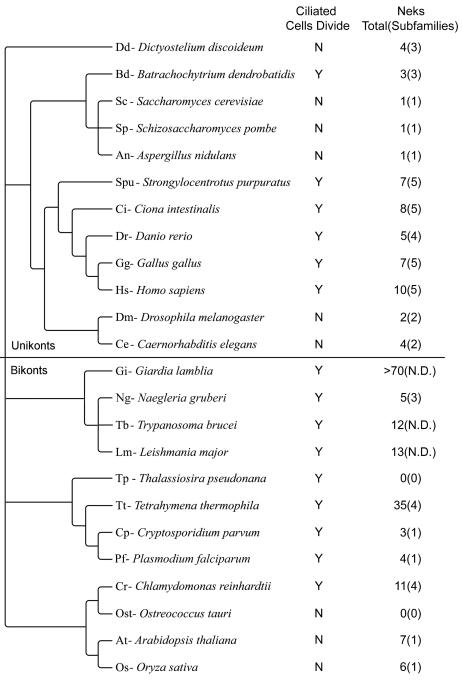
Cladogram of species included in our analysis. Relationships among taxa based on [29], [55]. The “Ciliated cells divide” column indicates presence (Y) or absence (N) of cells in the species which both bear cilia and re-enter the cell cycle: gametes and other terminally differentiated ciliated cells are excluded. The “Neks” column indicates both how many genes encoding Nek kinase domains were identified in the indicated species. The number in parentheses is a minimal estimate of how many Nek subfamilies are represented in the genome, as determined from our analysis (see text). N.D. indicates that we did not determine the number of subfamilies for the particular species.

Species from a broad range of eukaryotic lineages, with publicly-available sequenced genomes, were selected for analysis (Figure 1). We generated a dataset of Nek kinase domains from these species via the following methods: with the NIMA kinase domain as a query sequence, we used BLASTP analysis [30] to identify Neks from the predicted proteomes, using their publicly-available genomes. Top scoring BLASTP hits from the predicted proteomes were then reciprocally used as queries of the non-redundant NCBI GenBank database. Only those queries that produced Nek proteins as top scoring BLASTP hits were retained in the dataset. Only kinase domains, as predicted by the SMART HMMER search tool [31], [32] were included in our phylogenetic analysis, leading us to exclude the predicted human Nek10 (GenBank id NP_001026911), amongst other weak hits from various other organisms. Multiple sequence alignments were produced using ClustalX software [33] and manually inspected. Any kinase domain sequences containing large insertions or truncated kinase domains, likely the result of incorrect protein prediction, were excluded from the dataset at this stage. Our master list of kinase domains is available as supplemental data (Table S1).

In the course of our BLAST searching, we found some prokaryotic kinases which were reciprocal best hits with Neks. However, these sequences do not represent *bona fide* Neks, as they are outgroup to a clade containing multiple eukaryotic kinase families (not shown).

We used the Metropolis-coupled Markov chain Monte Carlo program MrBayes v. 3.1.2 [34], [35] to infer phylogenies. We allowed the program to determine the most probable model of protein evolution (aamodel), and specified an inverse gamma distribution of variability in substitution rate. Each analysis was composed of two simultaneous runs of either four or eight MCMC chains run for at least 2×10^6^ generations, sampled every 100 generations, and run until the average standard deviation of split frequencies between the two runs remained below the 0.01 convergence threshold for at least 1×10^5^ generations. Ninety percent of each run was burned in before the parameters were analyzed. Human Cdk2 was used to root all trees in either Treeview [36] or HyperTree [37]. HsCdk2 was selected as the outgroup because on a tree including Cdk2, Mlk3, AuroraA, Plk1 and the mammalian Neks, Cdk2 was the kinase most closely related to the Neks (data not shown). See Supplementary Table 1 for detailed tree statistics.

**Table 1:** Results of hypothesis-testing for the unikonts+*Chlamydomonas* dataset (Figure 2).

Topology Constraint	Num. of Gens. (×10^6^)	Avg. Std. Dev. of Split Freqs. Between Runs	Arithmetic Mean of log Likelihood Values	Harmonic Mean of log Likelihood Values	Bayes Factor
Unconstrained	3.00	0.008612	−22754.85	−22798.76	-
Node 4	5.53	0.005674	−22756.15	−22805.89	14.26
Node 10	5.51	0.006724	−22752.78	−22799.41	1.30
Node 9	4.34	0.006191	−22754.77	−22802.28	7.04
Node 7	3.90	0.004951	−22753.95	−22800.50	3.48
Node 6	2.58	0.008200	−22755.26	−22799.71	1.90
Node 3	3.47	0.007247	−22755.96	−22798.18	1.16
Node 4+Node 2	2.67	0.007250	−22813.60	−22861.38	125.24
Node 5+Node 2	2.33	0.008065	−22802.93	−22844.09	90.66
Ce D1044.8+Node 1	3.59	0.006875	−22756.55	−22802.11	6.70
Node 8+Node 7	4.01	0.006743	−22756.61	−22807.16	16.80
Node 5+Node 1	3.03	0.006723	−22767.36	−22820.18	42.84
Node 4+Node 1	5.18	0.007722	−22764.13	−22816.73	35.94
Node 4+Node 5	4.49	0.006659	−22755.90	−22798.78	0.04

**Figure 2:**
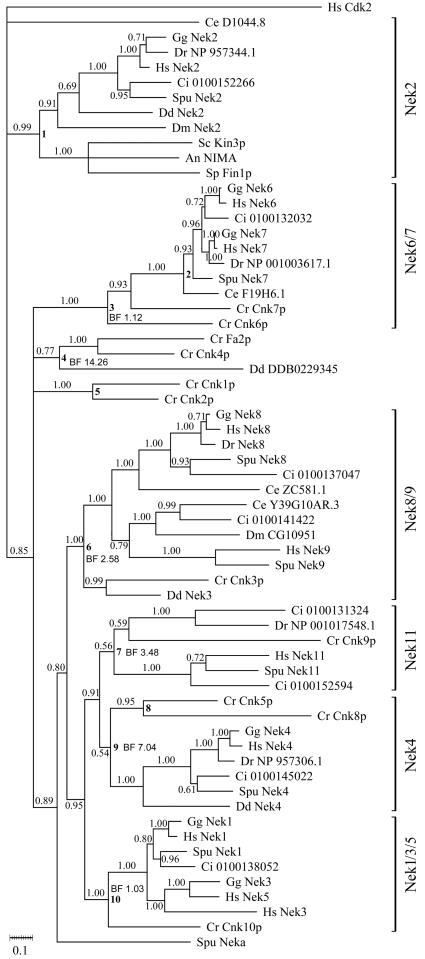
Phylogram of Nek kinase domains from unikonts+*Chlamydomonas.* Numbers at nodes are interpreted as *a posteriori* support values. Bold digits at some nodes indicate nodes used for hypothesis testing, and the Bayes Factor (BF) where indicated. Right brackets include sequences joined by well-supported internal nodes, which we propose to refer to as Nek subfamilies, named after the human member with the shortest branch length.

Using Bayes Factor analysis, values <10 suggest the constrained topology is a reasonable representation of the data [38], [39]. Constrained nodes are indicated in Figure 2; “Node×+Node y” indicates all taxa arising from both nodes were constrained to branch from a single internal node.

To test hypotheses on the unikonts plus *Chlamydomonas* dataset we first analyzed the unconstrained dataset and obtained a harmonic mean of the log likelihood values of the MCMC samples (Table 1). We then re-ran the analysis, constraining the topology to reflect various hypotheses (for example, constraining nodes or pairs of nodes, Table 1), and recorded the harmonic mean of log likelihood values. Twice the difference in mean harmonic likelihood (known as the Bayes Factor) can be used to determine relative support for alternative hypotheses; values for the Bayes Factors of >10 indicate strong support for the less constrained tree over the *a priori* imposed constraint (i.e. the hypothesis). [38], [39]. Analyses were otherwise run as described above. Constraining the amino acid model did not affect either the topology or likelihood of the resulting tree (Table 1).

## Results

We use the terminology of Cavalier-Smith [29] to refer to the major eukaryotic lineages: unikonts include Metazoa, Fungi, and Amoebozoa, while bikonts include all other organisms considered. We found Neks from a broad sampling of eukaryotic lineages (Figure 1), but we identified no Neks in the genomes of the diatom *Thalassiosira* or the green alga *Ostreococcus*.

Large numbers of Neks were found in the genomes of the kinetoplastids *Leishmania* and *Trypanosoma*, as well in the genome of *Giardia* (Figure 1). Inclusion of sequences from either the kinetoplastids or *Giardia* in our analyses lead to failure of convergence, so we excluded these species from further analysis. We note that these species are parasitic, and recent analyses consider them to be highly derived, rather than basal, eukaryotes [40]. This, in combination with their large number of Nek sequences, accounts for the difficulty in resolving Nek trees including these organisms. However, our analysis indicates that most of the kinetoplastid Neks form a lineage-specific expansion in a clade with human Nek8 ( Figure S1). Our SMART analysis showed that with rare exceptions (all of which are in *Tetrahymena*) all sequences recognized as having a Nek-like kinase domain had that domain at the N-terminus of the predicted protein; when C-terminal domains were predicted by SMART, these were always protein-protein interaction domains. These results are consistent with the view that Neks have rapidly-evolving C-terminal regulatory/interaction domains, allowing differential localization, regulation, or function in various lineages.

**Figure 3:**
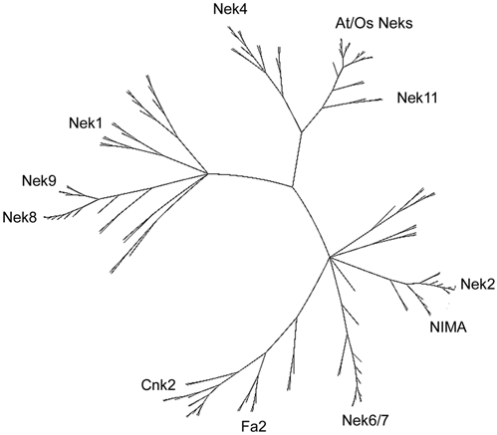
Phylogram of Nek kinase domains from a larger sampling of eukaryotes. Neks from all species shown in Figure 1 having Neks in their genome were included in this dataset except for *Leishmania*, *Trypanosoma*, and *Giardia*. This represents an overview of the tree which is examined in detail in Figures 4–7.

A dataset consisting of the unikont Neks plus the *Chlamydomonas* Neks was the largest dataset we could analyze in a reasonable time (∼1 week on a desktop computer), while inclusion of the bikont Neks to produce the tree in Figure 3 required much more analysis time (∼3 months) on a computer cluster using the equivalent of eighteen desktop processors. Due to these computational limitations, we performed stringent analyses by Bayesian testing of alternate topologies on the former dataset, represented in Figure 2 and Table 1 (see below).

The topology of the tree in Figure 2 is consistent with previous studies (e.g. [41]), but the inclusion of several additional species in our tree allows us to differentiate more clearly orthology and paralogy. For example, analysis of the unikont Neks (Figure 2) suggests that hNek2 is indeed the NIMA orthologue, based on the observations that the nodes connecting these taxa are strongly-supported (posterior probability typically ∼1), and contain sequences from all the unikont taxa included, including distantly-related taxa such as humans and yeast. Consequently, we refer to this group of sequences as the Nek2 clade. Similarly, we note that the human Neks are scattered among six *a posteriori*-defined clades, each of which contains representatives from most species tested and most of which have well-supported nodes (posterior probability >0.85, Figure 2). Importantly, the above statement is valid despite the inclusion of *Chlamydomonas*; that is, most *Chlamydomonas* Neks fall into the same well-supported clades as the Neks of the unikont species included in this analysis.

Four of the ten *Chlamydomonas* Neks are not members of any clades in Figure 2. In order to test whether this was a *bona fide* result, we tested various alternative hypotheses for the topology. We constrained Cnk1p and Cnk2p (node **5** in Figure 2) to be sister to the Nek2 clade (node **1** in Figure 2), and calculated the resulting Bayes Factor (Table 1, “node 5+node 1”). The Bayes Factor of 42 strongly rejects a composite Cnk2/Nek2 clade in favour of the topology seen in Figure 2. As controls, we repeated this analysis to test alternate topologies for the *Chlamydomonas* sequences which fell within well-supported clades in Figure 2. Reassuringly, the topology reported in Figure 2 was not rejected for any of these hypotheses, including Cnk10p membership in the same clade as vertebrate Nek1 (Table 1). We repeated this experiment for less well-supported nodes, e.g., the Nek4 and Nek11 clades. Cnk5p and Cnk8p are sister to unikont Nek4's with a posterior probability of only 0.54, but could not be rejected as members of the Nek4 clade by Bayes Factor Analysis (BF = 7.04); a similar result was obtained indicating that Cnk9p is a tentative member of the Nek11 clade.

In only three instances did Bayes Factor analysis reveal a lack of support for the tree reported in Figure 2: the *Dictyostelium* DDB0229345 sequence was rejected from the Fa2p/Cnk4p clade, the *C.elegans* sequence D1044.8 was rejected from the Nek2 clade, and the Cnk2p/Cnk1p and Fa2p/Cnk4p clades cannot be distinguished as independent clades (Table 1). In summary, the analyses presented in Figure 2 and Table 1 indicate at least five *bona fide* Nek clades with members from both the mammal *Homo* and the green alga *Chlamydomonas*, plus two additional clades, the *Chlamydomonas*-specific Fa2p/Cnk2p clade, and the unikont-specific Nek2 clade.

**Figure 4:**
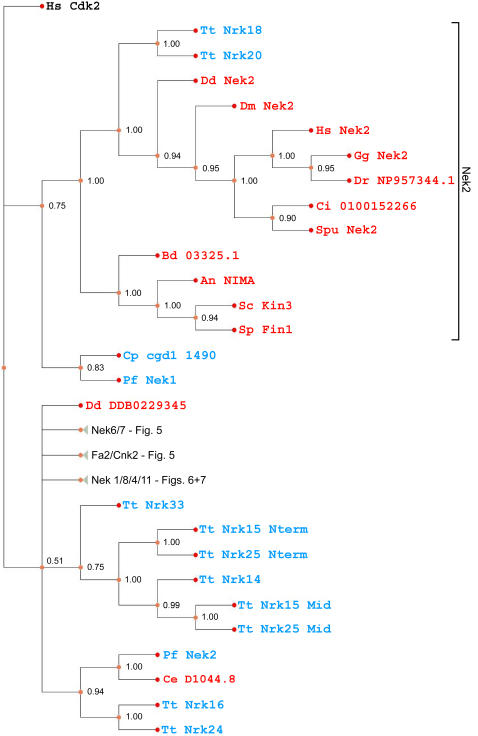
Phylogram of Nek kinase domains across eukaryotes. For ease of visualization, large nodes have been collapsed (triangles) and are shown expanded in subsequent figures (Figures 5–7) as indicated. In these figures sequences from bikont species are cyan, while sequences from unikont species are red. Right brackets include sequences joined by well-supported internal nodes, which we propose to refer to as Nek subfamilies, named after the human member with the shortest branch length where appropriate.

**Figure 5 pone-0001076-g005:**
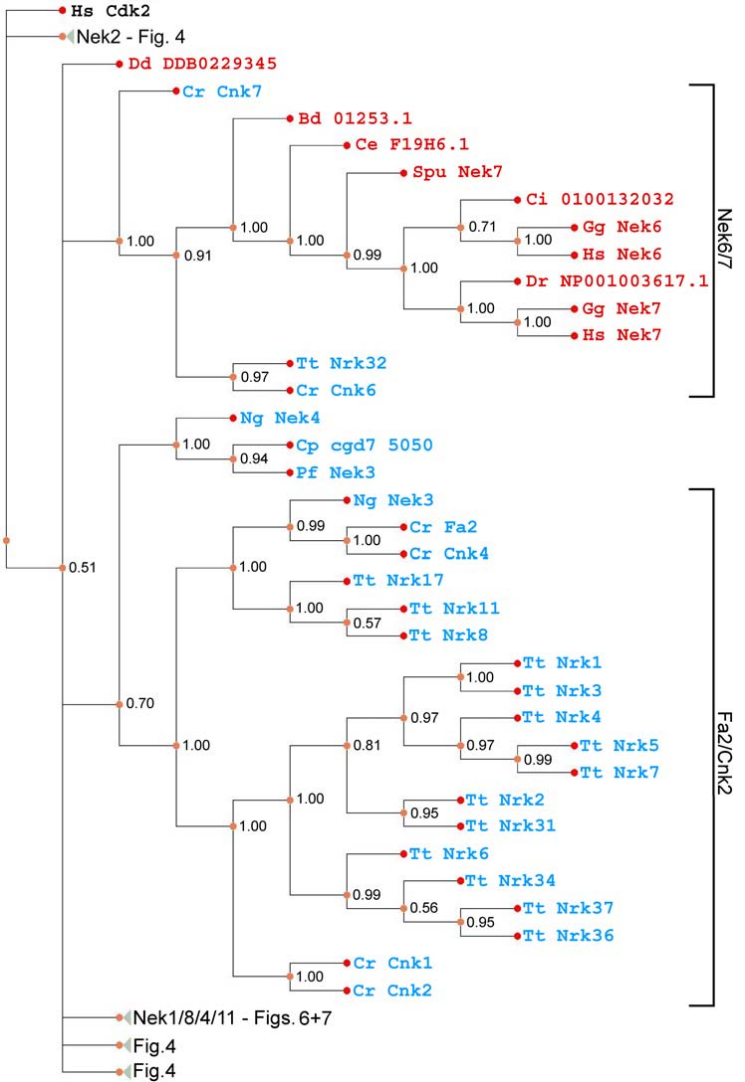
Phylogram of Nek kinase domains across eukaryotes. For details see legend to Figure 4.

**Figure 6 pone-0001076-g006:**
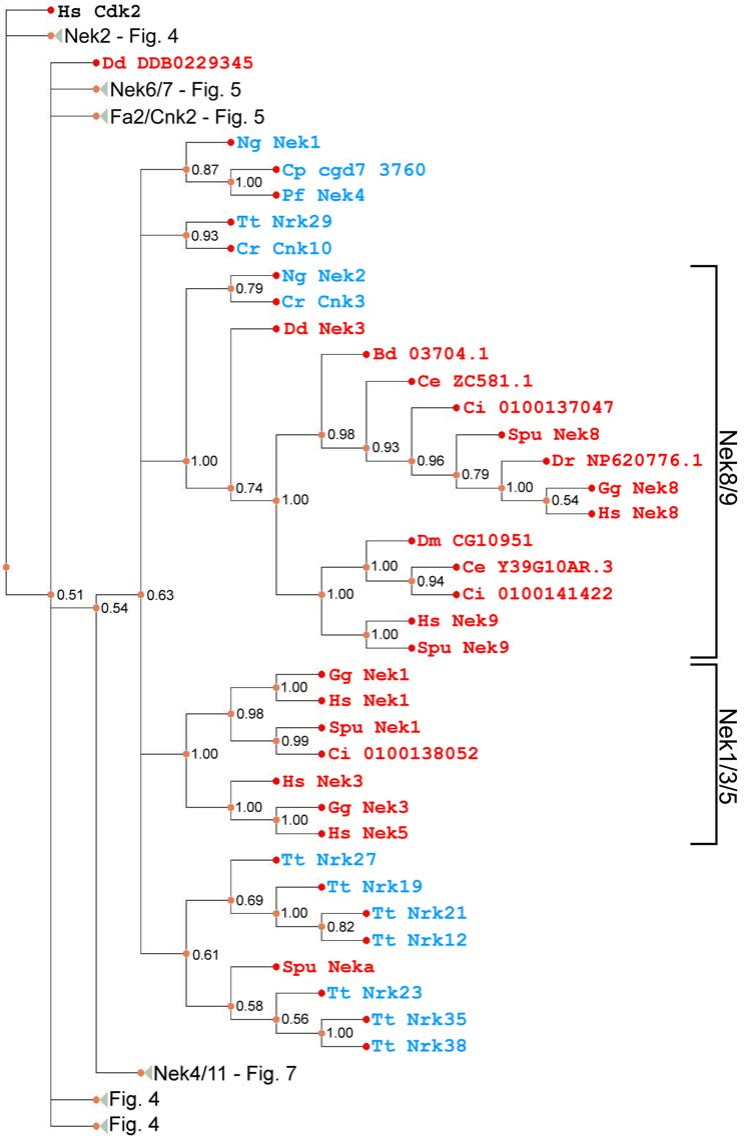
Phylogram of Nek kinase domains across eukaryotes. For details see legend to Figure 4.

Addition of Nek sequences from other bikonts, as well as the (unikont) chytrid fungus *Batrachochytrium* to the data set used to generate Figure 2 did not significantly affect the topology of the tree (Figure 3). Several of the newly-added sequences arise in the tree as polytomies, a result consistent with the difficulties inherent in constructing a multigene phylogeny across a broad sampling of eukaryotes. However, with the exception of the Nek1/3/5 clade, but including the Nek2 and Cnk2p/Fa2p clades, our previously-defined clades now include sequences from distantly related species such as *Arabidopsis*, *Naegleria*, and *Tetrahymena* (Figures 4–7). The previously unikont-only Nek2 clade has members from *Tetrahymena*, while two apicocomplexan sequences (Cp cgd1 1490 and Pf Nek1) branch near the well-supported Nek2 clade (Figure 4). The Nek6/7 clade is well-supported, and like the Nek2 clade, now contains sequences from *Tetrahymena* and *Batrachochytrium* (Figure 5). Satisfyingly, Cnk2p and Fa2p now form a clade with several *Tetrahymena* Neks (consistent with Wloga et al. [26]), as well as a *Naegleria* Nek (Figure 5). Similarly, the Nek8/9 clade remains intact (Figure 6), and now contains *Naegleria* “Nek2”, which has a similar C-terminal domain composition to its orthologue in *Chlamydomonas*, Cnk3p (not shown).

**Figure 7:**
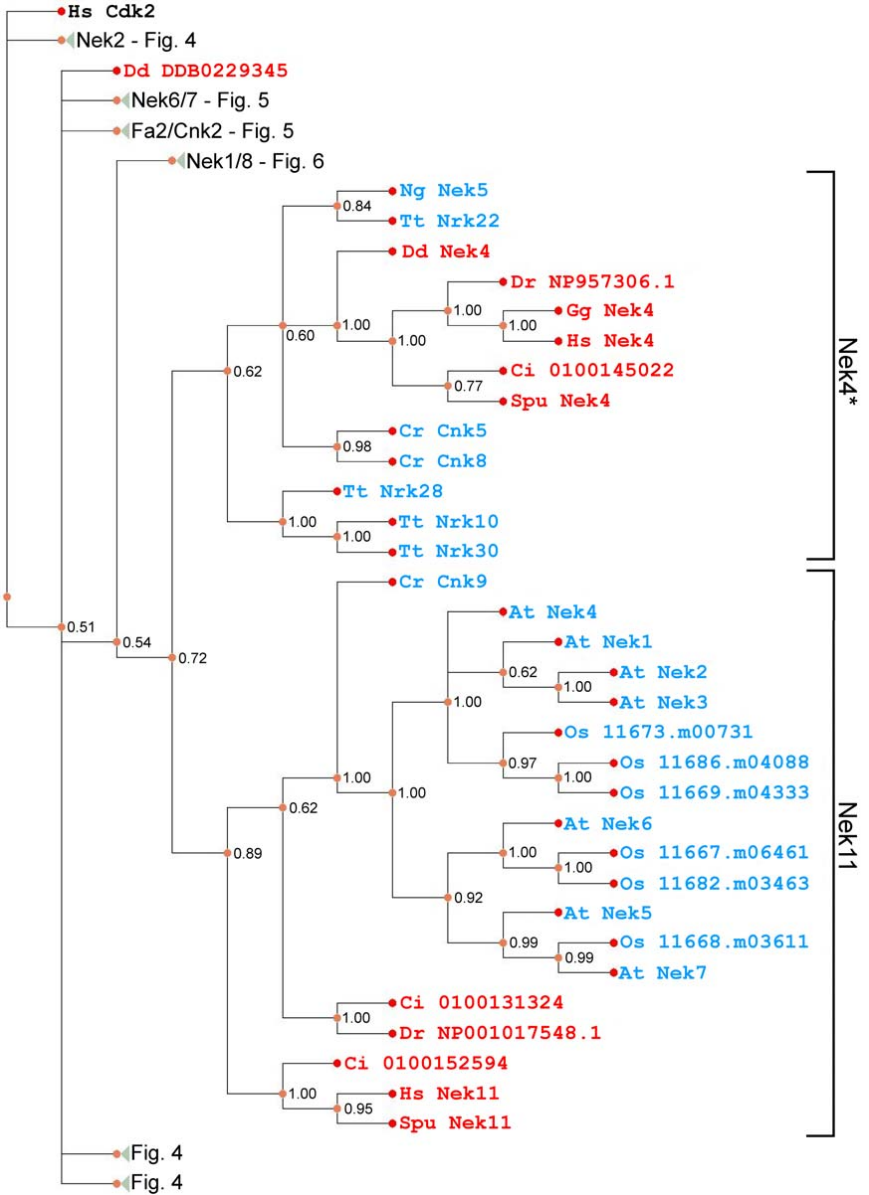
Phylogram of Nek kinase domains across eukaryotes. For details see legend to Figure 4. The Nek4 clade is marked with an asterisk due to its poor support (see text).

The Nek1/3/5 clade did not resolve with sequences from both unikonts and bikonts (Figure 6). However, a tree from a smaller dataset (Figure S2) shows that *Chlamydomonas* Cnk10p, *Tetrahymena* Nrk29 and *Cryptosporidium* cgd7 3760 are members of a well-supported Nek1/3/5 clade (posterior probability of 0.94). This suggests that some of the clades arising from the polytomy could be orthologous to members of the unikont Nek1/3/5 clade. Above, we provided additional evidence that Cnk10p is a Nek1/3/5 family member (Figure 2, Table 1, Figure S1, Figure S2). Unfortunately, tests of hypotheses on this larger dataset are not feasible with our current methods and available computational power.

Consistent with our previous results (Figure 2 and Table 1), the Nek4 and Nek11 clades are closely related to each other and thus difficult to resolve (Figure 7). We note that human Nek11 (a protein localized to the nucleolus [9]) and the higher plant Neks are all in the Nek11 clade, consistent with the hypothesis that a non-centrosomal Nek family expanded in a lineage which does not have centrosomes; however, these Neks still play roles in cell cycle regulation [42], [43]. In contrast, we find *Tetrahymena* Nrk30p, which localizes to cilia [26], to be in the Nek4 clade (Figure 7). We therefore hypothesize that in contrast to the Nek11 clade, members of the Nek4 group may retain ciliary function.

Neks were found in nearly every genome sampled, and the numbers of Neks we identified roughly correlates with whether the organism has non-terminally differentiated ciliated cells (Figure 1). We included both the raw number of Neks found in the organisms' genomes, and the minimal number of Nek subfamilies represented, to further clarify the Nek/cilia relationship. We define our Nek subfamilies as moderately well-supported clades (posterior probability >0.85) with members from both unikont and bikont species. The Nek2, Nek6/7, Nek8/9, and Nek11 clades meet this definition (Figures 4–7). As discussed above, the Nek1/3/5 clade is well-supported in Figure S2. Also, the Nek 11 clade in Figure 7 meets our definition, but the Nek4 clade does not; however, a composite Nek4/11 clade is well-supported in our smaller dataset (Figure S2). Thus, we define five Nek subfamilies, named after their human members: Nek1/3/5, Nek2, Nek4/11, Nek6/7, and Nek8/9. Our analysis thus allows identification of lineage-specific expansions, for example, the plant *Arabidopsis* has seven Neks in its genome, but all seven are part of an expansion in the same Nek subfamily (Figures 3). Conversely, *Batrachochytrium* has only four Neks, but these all belong to separate subfamilies. Thus a correlation is evident amongst the organisms sampled between the presence of ciliated cells which divide and the diversity of Neks (Figure 1).

## Discussion

We draw two major conclusions from our phylogenetic analysis. First, tree reconciliation suggests that the last common ancestor (LCA) of unikonts and bikonts most likely had at least five Neks and variations in the numbers of Neks in different lineages was due to expansion or loss of multiple Neks; this is a novel finding and changes our view of this kinase family and the relationships of its members. Second, this work confirms and extends our previous suggestion that Neks are more abundant in the genomes of organisms which must co-ordinate ciliary assembly and disassembly with respect to the cell cycle.

Currently, there is a paucity of functional data relevant to the cell biology of the Nek family. Consequently, there is insufficient data available to discern clear roles for particular Nek subfamilies from comparison of the Nek repertoires. However, the availability of the relationships between the various Neks will make future research on any particular Nek more relevant to the field at large. While most Neks that have been studied are associated with cilia/centrosomes/cell cycle, we do not propose that all Neks subserve this triumvirate. Indeed, expansion of the subfamilies was inevitably associated with both loss of function and acquisition of new roles [44], but analysis of which Neks have ancestral functions and which have derived functions requires the availability of phylogeny such as we present here. For example, Nek3, which is not centrosomal and has functions seemingly unrelated to microtubular-based structures [45], [46], is now revealed to be a relatively recent innovation (Figure 2), which likely underwent neofunctionalization.

Although we cannot discern cellular roles for individual Nek subfamilies, it is clear that Neks are not required for the formation of centrioles or cilia. Diatoms, for example, make both of these organelles *de novo* when differentiating into gametes [47] but have no Neks. Nor are the Neks required for mitosis, as exemplified by the many species whose genomes do not encode Neks. Importantly, we have yet to find a species that does not encode any Neks but does have ciliated cells that divide. Conversely, the *Giardia* genome has >70 Neks; *Giardia* flagella undergo a complex maturation process which takes four cell cycles [48], which may represent an extreme case of requirement for Neks during division in a flagellated cell. We predict that as functional studies progress, it will be revealed that many Neks participate in the important cellular role of coordinating the activities of centrioles as basal bodies during interphase and as spindle pole foci during mitosis.

Given our phylogenetic results, ciliary and centrosomal Nek functions are likely to be ancestral and thus may be retained in phylogenetically distant organisms. The wide distribution of the Neks from both unikonts and bikonts suggests strongly that the Neks had already expanded in the eukaryotic LCA. We suggest deviations of the topologies of the Nek subfamilies from our analysis from the species tree may represent different evolutionary constraints in the various lineages.

Our conclusion that the LCA, which must have been a non-terminally differentiated ciliated cell, had at least five Neks, should be interpreted in two ways: first, we are discerning the nature of the eukaryotic LCA, which is distinct from the first eukaryote; second, the presence of several Neks in the LCA conforms well with our idea that this kinase family is ancestrall*y* correlated with the presence of ciliated cells which divide. The cell biology of the eukaryotic LCA can be partially inferred by applying the principal of parsimony to diverse extant eukaryotes: it is thus likely that the LCA was ciliated with a 9+2 axoneme and centrioles with triplet microtubules, and that the LCA could undergo both mitosis and meiosis [49]. The feature of extant eukaryotes most predictive for the number of retained Nek subfamilies appears to be the presence of ciliated cells which can re-enter the cell cycle (Figure 1 and [2]). Our data is consistent with a growing body of data which suggests that the eukaryotic last common ancestor had a sophisticated cellular organization and possessed many proteins required for centriole/cilium assembly [50], [51]. It is also consistent with the notion that early eukaryotes underwent a period of rapid evolution, especially of cytoskeletal elements [52]. We suggest that the Neks may have expanded during this time.

Although the relationship between cilia and the cell cycle is a long-standing problem in cell biology [53] which is receiving renewed interest [27], [54], our understanding of the complex regulatory relationship that must exist remains rudimentary. Functional studies of the Nek family may provide new points of entry into this important but undoubtedly complex signaling network.

## Supporting Information

Table S1 (0.03 MB DOC)

Figure S1 Phylogram of the largest and most diverse dataset containing Leishmania and Trypanosoma sequences that was able to converge. This tree is rooted on HsCdk2. Please note that the majority of Leishmania and Trypanosoma Neks are members of a well-supported clade that includes HsNek8 (posterior probability = 0.90).
(1.05 MB TIF)

Figure S2 Phylogram of Nek kinase domains from species listed in Figure 1 with the exception of Batrachochytrium, Plasmodium, Naegleria, Arabidopsis, and Oryza. This tree is rooted on HsCdk2.
(1.16 MB TIF)
