# Supplementary material for: Phylogenetic Analysis of the Neks Reveals Early Diversification of Ciliary-Cell Cycle Kinases
Source: PLoS One. 2007 Oct 24;2(10):e1076. doi: 10.1371/journal.pone.0001076 (PMC2031824; doi:10.1371/journal.pone.0001076)
Supplement: Table S1 — (0.03 MB DOC) [file pone.0001076.s001.doc]

Table S1. Detailed MrBayes output for datasets analyzed in this study.

| Dataset | Number of taxa | Number of chains/run | Num. of Gens. (x10^6^) | Avg. Std. Dev. of Split Freqs. Between Runs | Arithmetic Mean of log Likelihood Values | Harmonic Mean of log Likelihood Values |
| --- | --- | --- | --- | --- | --- | --- |
| Figure 2 | 63 | 4 | 3.00 | 0.008612 | -22754.85 | -22798.76 |
| Figures 3-7 | 128 | 8 | 16.39 | 0.009892 | -45555.61 | -45636.61 |
| Figure S1 | 78 | 8 | 4.88 | 0.008434 | -29007.99 | -29053.90 |
| Figure S2 | 101 | 8 | 7.21 | 0.009652 | -31078.76 | -31099.13 |
