# Supplementary figures and images for: Phylogenetic Analysis of the Neks Reveals Early Diversification of Ciliary-Cell Cycle Kinases
Source: PLoS One. 2007 Oct 24;2(10):e1076. doi: 10.1371/journal.pone.0001076 (PMC2031824; doi:10.1371/journal.pone.0001076)

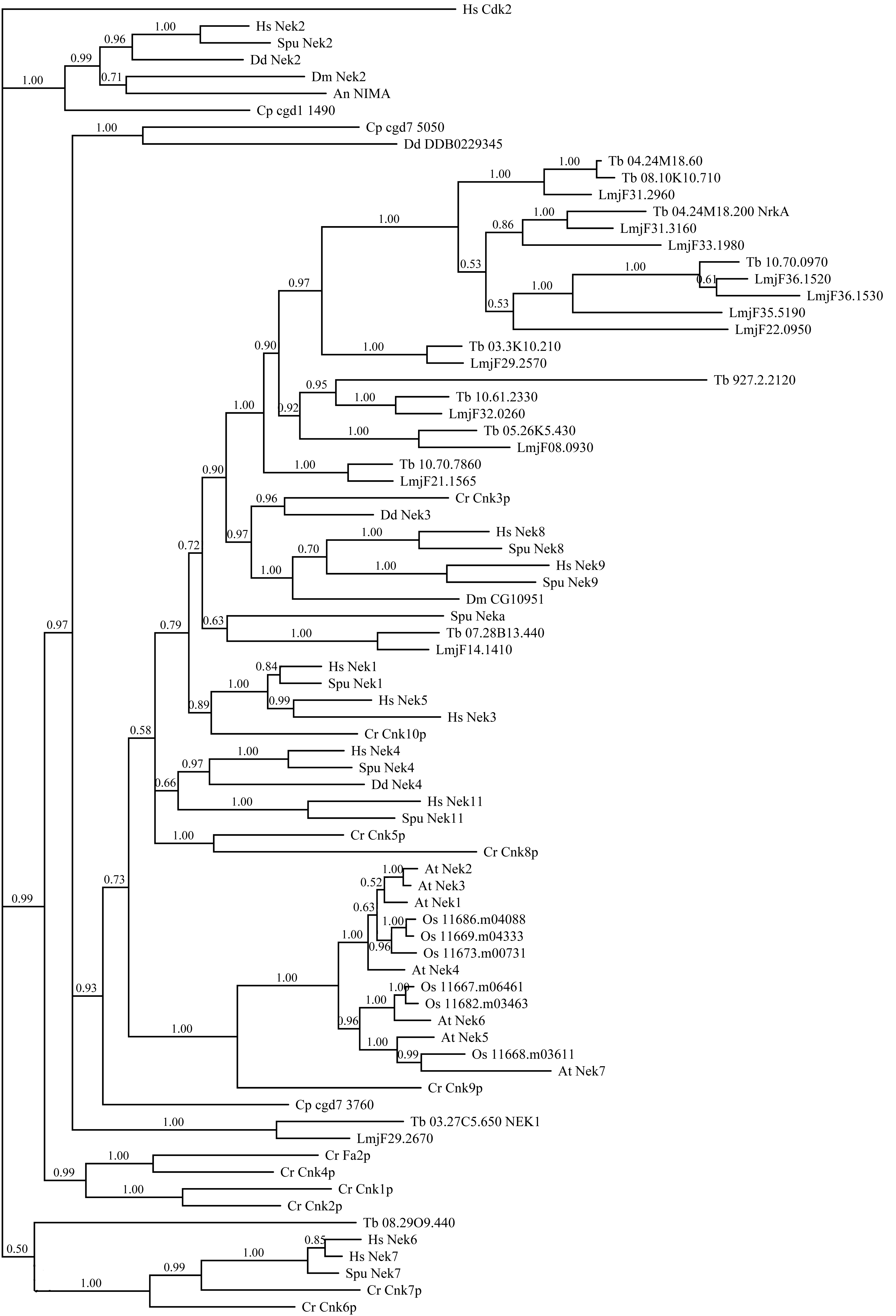

Supplement: Figure S1 — Phylogram of the largest and most diverse dataset containing Leishmania and Trypanosoma sequences that was able to converge. This tree is rooted on HsCdk2. Please note that the majority of Leishmania and Trypanosoma Neks are members of a well-supported clade that includes HsNek8 (posterior probability = 0.90). (1.05 MB TIF) [file pone.0001076.s002.tif]

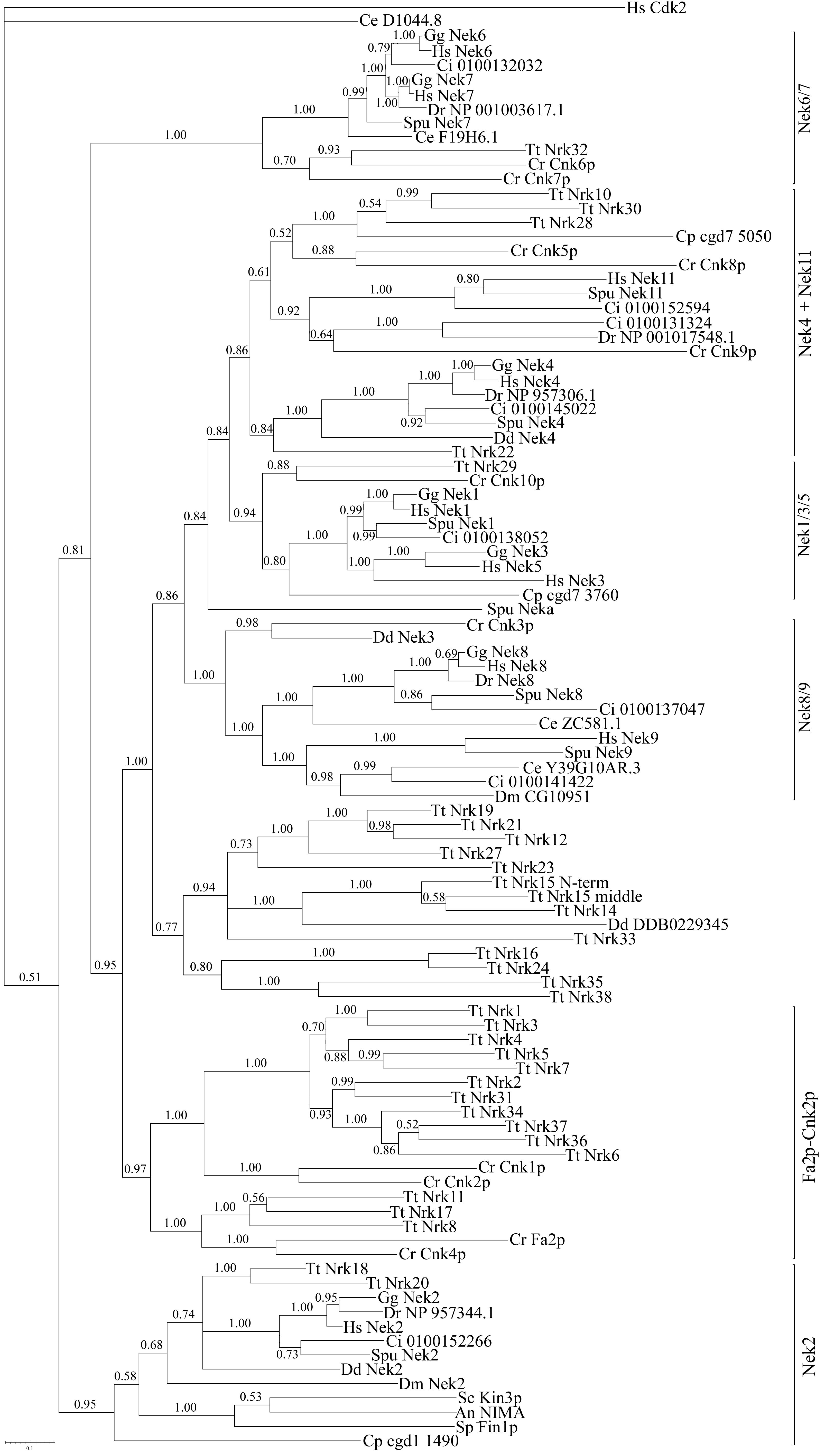

Supplement: Figure S2 — Phylogram of Nek kinase domains from species listed in Figure 1 with the exception of Batrachochytrium, Plasmodium, Naegleria, Arabidopsis, and Oryza. This tree is rooted on HsCdk2. (1.16 MB TIF) [file pone.0001076.s003.tif]
